# The Parental Non-Equivalence of Imprinting Control Regions during Mammalian Development and Evolution

**DOI:** 10.1371/journal.pgen.1001214

**Published:** 2010-11-18

**Authors:** Reiner Schulz, Charlotte Proudhon, Timothy H. Bestor, Kathryn Woodfine, Chyuan-Sheng Lin, Shau-Ping Lin, Marine Prissette, Rebecca J. Oakey, Déborah Bourc'his

**Affiliations:** 1 Department of Medical and Molecular Genetics, King's College London, London, United Kingdom; 2 Institut Curie, INSERM U934/CNRS UMR3215, Paris, France; 3 Department of Genetics and Development, Columbia University, New York, New York, United States of America; 4 Transgenic Animal Facility, Herbert Irving Comprehensive Cancer Center, Columbia University, New York, New York, United States of America; 5 Institute of Biotechnology, National Taiwan University, Taipei, Taiwan; 6 Department of Pathology, Columbia University, New York, New York, United States of America; The Babraham Institute, United Kingdom

## Abstract

In mammals, imprinted gene expression results from the sex-specific methylation of imprinted control regions (ICRs) in the parental germlines. Imprinting is linked to therian reproduction, that is, the placenta and imprinting emerged at roughly the same time and potentially co-evolved. We assessed the transcriptome-wide and ontology effect of maternally versus paternally methylated ICRs at the developmental stage of setting of the chorioallantoic placenta in the mouse (8.5dpc), using two models of imprinting deficiency including completely imprint-free embryos. Paternal and maternal imprints have a similar quantitative impact on the embryonic transcriptome. However, transcriptional effects of maternal ICRs are qualitatively focused on the fetal-maternal interface, while paternal ICRs weakly affect non-convergent biological processes, with little consequence for viability at 8.5dpc. Moreover, genes regulated by maternal ICRs indirectly influence genes regulated by paternal ICRs, while the reverse is not observed. The functional dominance of maternal imprints over early embryonic development is potentially linked to selection pressures favoring methylation-dependent control of maternal over paternal ICRs. We previously hypothesized that the different methylation histories of ICRs in the maternal versus the paternal germlines may have put paternal ICRs under higher mutational pressure to lose CpGs by deamination. Using comparative genomics of 17 extant mammalian species, we show here that, while ICRs in general have been constrained to maintain more CpGs than non-imprinted sequences, the rate of CpG loss at paternal ICRs has indeed been higher than at maternal ICRs during evolution. In fact, maternal ICRs, which have the characteristics of CpG-rich promoters, have gained CpGs compared to non-imprinted CpG-rich promoters. Thus, the numerical and, during early embryonic development, functional dominance of maternal ICRs can be explained as the consequence of two orthogonal evolutionary forces: pressure to tightly regulate genes affecting the fetal-maternal interface and pressure to avoid the mutagenic environment of the paternal germline.

## Author Summary

In mammals, a subset of genes is expressed from only one chromosomal copy, depending on its parental origin. This process, known as genomic imprinting, results from DNA methylation marks deposited in gametes at regulatory sequences called imprinting control regions (ICRs). Most of the DNA methylation controlling imprinting is established in the oocyte, while very few ICRs are methylated in the sperm. We provided insight into the impact and origins of the parental imbalance in genomic imprinting control. We defined the transcriptome-wide effect of imprinting, during the transition period when the embryo becomes dependent upon maternal resources. We found that maternal ICRs have a vital effect on developmental pathways related to the mother-to-fetus exchanges, while paternal ICRs have a dispersed and non-significant effect at that stage. We evidenced that paternal ICRs are lost at a much faster rate than maternal ICRs during mammalian evolution, probably as a mechanistic consequence of different kinetics of the parental germlines. Our results support the notion that two independent evolutionary forces have led to the numerical and functional dominance of maternal ICRs: a selective advantage of parent-specific regulation of genes important for the fetal-maternal interface and pressure to avoid the mutagenic environment of the paternal germline.

## Introduction

Mammalian development requires a subset of genes to be expressed in a parent-of-origin manner at specific stages and in specific tissues [1]. These so-called imprinted genes are organized around *cis-*acting regulatory sequences termed imprinting control regions (ICRs) that display allele- and parent-specific DNA methylation. The parental determinism results from the sex-specific acquisition of these methylation marks, or imprints, on maternal and paternal alleles during gametogenesis [2] by the combined action of *de novo* DNA cytosine-5-methyltransferases and their co-factor DNMT3L [3], [4]. By convention, the term maternally or paternally imprinted gene refers to the parental origin of the methylation mark targeting the associated ICR, but does not specify from which parental allele the gene is expressed.

While *de novo* methylation of parental ICRs invariably coincides with periods of developmental quiescence both in female and male gametogenesis, the biology of maternally versus paternally methylated ICRs differs significantly [5], [6]. *De novo* methylation of maternal ICRs is a post-meiotic event that occurs after birth in cohorts of growing oocytes. Methylation of paternal ICRs takes place prior to meiosis, in fetal male germ cells [7]. Both the number and density of methylation targets, that is, CpG dinucleotides, are high at maternal ICRs, which always coincide with promoters. In contrast, paternal ICRs map to intergenic regions of relatively low CpG content. Finally, while roughly equal numbers of imprinted genes are either maternally or paternally expressed, ICR methylation is mainly of maternal origin [8]. More than 16 ICRs inherit their methylation from the oocyte, while only 3 ICRs carry methylation transmitted by the sperm (*H19/Igf2*, *Gtl2/Dlk1* and *Rasgrf1* loci). A fourth locus bearing paternal germline methylation has been recently described, the *Gpr1/Zdbf2* locus, but its regulatory role on imprinted expression is unknown [9].

The above differences between maternal and paternal ICRs are accompanied by an asymmetric influence on mammalian development. Pioneering work in constructing uniparental conceptuses by nuclear transfer in the mouse showed that parthenogenetic embryos with two maternal genomes died before 8.5dpc (days *post-coitum*) with severely reduced extraembryonic structures, while diploid androgenetic embryos of strictly paternal origin died earlier, with a small embryonic contribution and hyperproliferative extraembryonic structures [10], [11]. However, nuclear transplantation studies cannot define the net influence of maternal and paternal imprints on development because these create two sets of either maternal or paternal genomes, with a compounding effect of imprint excess of one parental origin and lack of imprints from the other parent. Next generation models of imprinting deficiency demonstrated the earlier requirement of maternal imprints for development: a specific lack of maternal imprints compromises embryonic viability at 9.5dpc, while the absence of paternal germline imprints leads to a later lethality, at 13.5dpc [3], [6], [12]. In both cases, the development of extraembryonic tissues is severely altered, in agreement with the proposed evolutionary link between placentation and genomic imprinting in eutherian mammals [13]. However, despite the key role of genomic imprinting for mammalian physiology, the overall effects that maternal and paternal imprints exert on the early embryo transcriptome are unknown, especially at the key developmental time when placentation and vascularization occur (around 8dpc in mouse). This stage represents a crucial transition, where after a period of autonomous growth, the continued embryonic development becomes strictly dependent on maternal resources allocation. Paternal imprints do not seem to be essential for the early embryo to make this transition, but it cannot be excluded that they exert some effects at this stage that will only become apparent later, at 13.5dpc.

Here, we gain insight into the importance of genomic imprinting for the early mammalian embryo (8.5dpc) by a functional dissection of the global gene regulatory impact of maternal versus paternal ICRs at the time of establishment of the fetal-maternal interface through the chorioallantoic placenta. Biological processes under the control of maternal versus paternal ICRs were defined by comparing the transcription profiles of fully imprinted embryos versus maternal imprint-free and completely imprint-fee embryos derived from *Dnmt3L* mutant mice. Overall, we found that maternal and paternal ICRs have a similar quantitative impact on the transcriptome of the early embryo. However, at 8.5dpc, only the effects of maternal ICRs were focused on biological pathways related to the fetal-maternal interface. In contrast, paternal ICRs elicited, in terms of biological processes, a broad and shallow effect.

We previously hypothesized that the different methylation histories of the two parental germlines may underlie the numerical imbalance between maternal and paternal ICRs [5], [6]. Deamination of 5-methylcytosine occurs at a 10-fold higher rate than other transitions, leading to frequent CpG to TpG/CpA mutations in mammalian genomes despite a dedicated repair pathway [14]–[17]. Here, we test this hypothesis by a systematic assessment of the sequence evolution of ICRs in different mammalian lineages and in comparison to other sequence categories. In doing so, we provide evidence that paternal ICRs have lost CpG sites and therefore their methylation targets at a significantly higher rate than maternal ICRs, while the latter in fact exhibit a relative gain of CpG motifs compared to similar but non-imprinted genomic regions. We propose that a combination of high mutational pressures at paternal ICRs together with functional selective pressure reinforcing methylation-dependent repression of ICRs, has led to the oocyte dominating the control of the fetal-maternal interface through genomic imprinting in eutherian mammals. Our results provide the first comprehensive view of the forces acting upon the regulatory sequences governing genomic imprinting in mammals.

## Results

### Developmental and epigenetic characterization of imprint-free embryos

The impact of imprinted gene expression on development prior to mid-gestation has never been investigated on a genome-wide scale. To understand which biological pathways are regulated by maternal and paternal ICRs, respectively, we compared the developmental potential and transcription profiles of 8.5dpc embryos that differ in their imprinting status but have an otherwise normal genome. Three different imprinting states were investigated: fully-imprinted (MP) embryos, maternal imprint-free (0P) embryos and completely imprint-free (00) embryos. Here, M and P denote a normally imprinted set of respectively maternal and paternal chromosomes, and 0 denotes a chromosome set without imprints.

**Figure 1:**
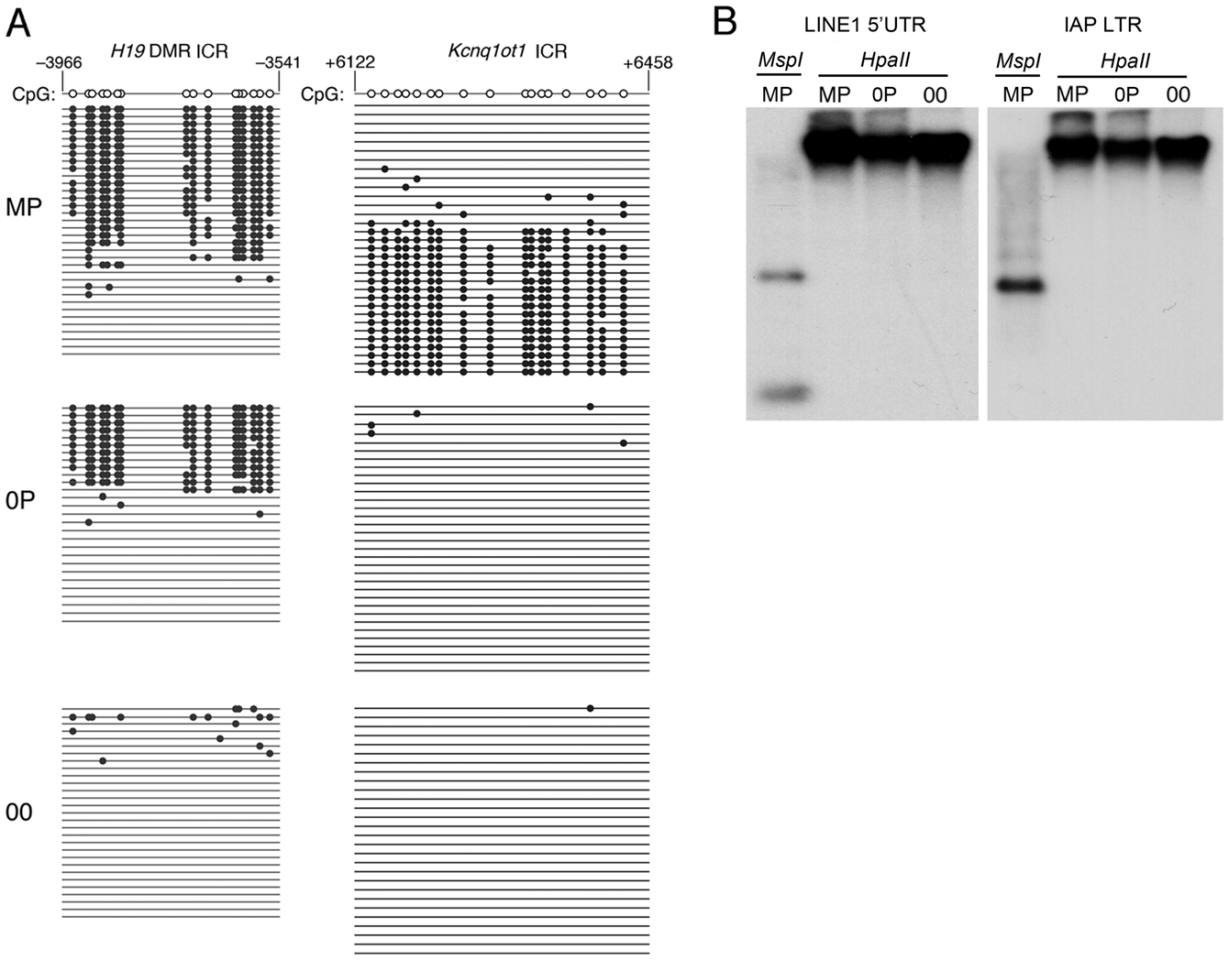
Methylation patterns in maternal-imprint free 0P and complete-imprint free 00 embryos at 8.5dpc. *A*, Bisulfite genomic sequencing of ICRs associated with the *H19* and *Kcnq1ot1* loci. The paternally methylated *H19* ICR was methylated in wildtype (MP) and in maternal imprint-free (0P) but unmethylated in imprint-free (00) visceral yolk sacs. The maternal *Kcnq1ot1* ICR was hypomethylated in both 0P and 00 material, in agreement with the lack of maternal imprints in these embryos. Nucleotide positions are reported in reference to the gene transcription start (+1). *B*, Normal methylation of LINE1 and IAP retrotransposons in MP, 0P, and 00 8.5dpc embryos as established by DNA blot hybridization after cleavage with the methylation-insensitive restriction endonuclease *MspI* or the methylation sensitive isoschizomer *HpaII*.

**Figure 2:**
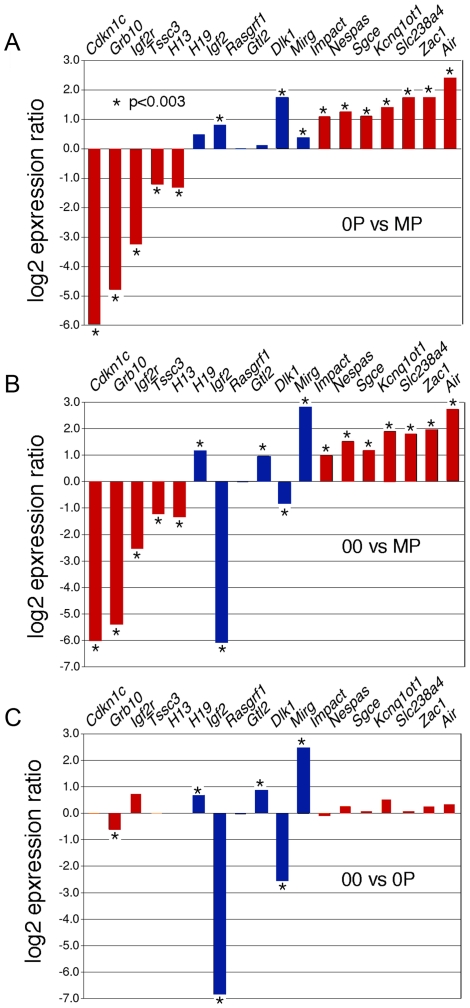
Expression abnormalities at imprinted loci in 8.5dpc 0P and 00 embryos. Microarray-measured gene expression ratios for known imprinted genes under the control of either maternally (red) or paternally (blue) methylated ICRs. The absolute expression levels were calculated using GC-RMA. Asterisks indicate a significant difference in expression (GCOS p-value <0.003). *A*, Imprinted gene expression in maternal imprint-free 0P embryos relative to wildtype biparental MP embryos. Maternally repressed imprinted genes (*Airn*, *Zac1*…) were upregulated, while maternally expressed imprinted genes (*Cdkn1c, Grb10*…) were downregulated. Changes observed at the paternally imprinted *H19*, *Igf2* and *Dlk1* genes are potentially secondary to the upregulation of *Zac1,* which is known to regulate the expression of multiple genes acting within the same functional network [19]. *B,* Imprinted gene expression in 00 imprint-free relative to MP embryos. As expected, maternally imprinted genes are as affected in 00 and 0P embryos compared to MP embryos. Genes controlled by paternal ICRs show changes expected to occur in the absence of paternal imprints, *i.e.* an upregulation of *H19* and *Gtl2* and subsequent downregulation of *Igf2* and *Dlk1*. *C,* In this comparison of 00 and 0P embryos, the significant changes in the expression of paternally imprinted genes persist, while for most maternally imprinted genes, no significant differential expression is observed, as is expected since both samples lack maternal germline imprints.

Diploid 0P and 00 embryos were obtained respectively by fertilization and artificial activation of maternal imprint-free oocytes carrying null alleles of *Dnmt3L*, a germline imprinting factor [3], [18]. To validate our approach, we initially confirmed the epigenotype of our embryonic models of imprinting deficiency, in particular of 00 embryos which have not been analyzed previously and should be maternal imprint-free, as the result from the *Dnmt3L* mutation, and paternal imprint-free, because of the lack of a paternal genome. Methylation analyses at the *H19* and *Kcnq1ot1* ICRs of 8.5dpc embryos revealed that 00 embryos lacked both maternal and paternal imprints, while 0P embryos specifically lacked maternal imprints (Figure 1A). Other genomic sequences were not affected. In particular, retrotransposons of the IAP and LINE-1 classes showed similar methylation levels in MP, 0P and 00 embryos (Figure 1B). Microarray analysis of imprinted gene expression showed that, as expected, genes controlled by maternal ICRs were significantly misexpressed in 0P and 00 embryos compared to MP embryos, while genes under the control of paternal ICRs were specifically misexpressed in 00 embryos compared to MP and 0P embryos (Figure 2 and Figure S1). In addition, the 0P versus MP comparison revealed a number of paternally imprinted genes significantly affected by the lack of maternal imprints (Figure 2A). Overxepression of the maternally imprinted *Zac1* gene has been previously shown to increase transcription of the paternally imprinted *H19*, *Igf2* and *Dlk1* genes in cellular assays, through a functional network linked to the control of embryonic growth [19]. We observed the exact predicted changes of expression of *H19*, *Igf2* and *Dlk1 in vivo*, as a result of *Zac1* upregulation by bi-allelic expression in maternal imprint-free 0P embryos. While we found that maternal ICRs act upstream of some genes under the control of paternal ICRs, the 00 versus 0P comparison showed that the reverse effect is comparatively small (Figure 2C). As a whole, methylation and expression analyses confirmed that genuine imprint-free 00 embryos had been obtained and differed from 0P embryos only by abnormal expression of paternal imprinted genes. The lack of a paternal genome in 00 embryos is unlikely to have any other major effect than the ones linked to imprinting, as animals carrying two maternal genomes and a genetic restoration of paternal imprints are viable [20].

**Figure 3:**
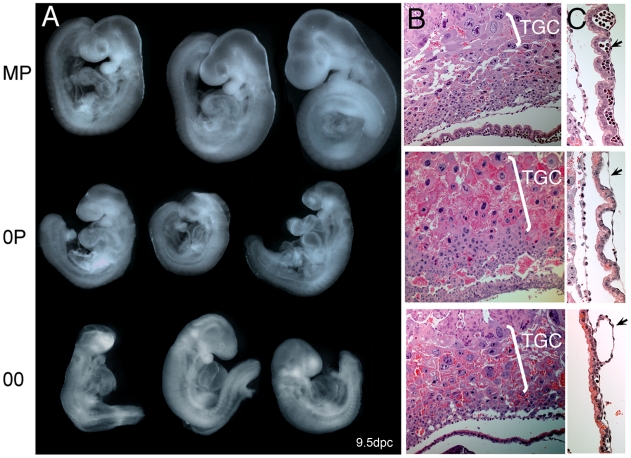
Net effects of loss of paternal and maternal germline imprints on early mouse development. *A*, Gross morphology of 9dpc MP, 0P, and 00 embryos. Note that 0P and 00 embryos are very similar in size and phenotype, which implies a minor contribution of paternal imprints to early development. Especially notable signs are the intrauterine growth retardation, open anterior neural tube, enlarged pericardium, reduced head size and abnormal craniofacial features. *B*., Hyperplasia of the trophoblast giant cell layer (TGC) in 0P and 00 conceptuses. *C.*, Severe deficiency in the vascularization (arrows) of 0P and 00 visceral yolk sacs. Note that erythrocytes are present in MP but are largely absent in 0P and 00 VYS.

Phenotypic analysis revealed that 00 and 0P embryos were developmentally similar at 9dpc (Figure 3). These embryos successfully progress through gastrulation and organogenesis but all cease development at around 8.5dpc, as revealed by examination of 00 embryos at later stages (Figure S2). The molecular defects associated with a lack of imprinting are multigenic. The phenotypic presentation may therefore be slightly variable from one embryo to the other, but recurrent signs were nonetheless observed. Intrauterine growth retardation and other signs of nutritional deprivation (swollen pericardial sacs and hemorraghe) were characteristics of both 00 and 0P embryos. These developmental abnormalities can be explained by defective chorioallantoic fusion, trophoblast giant cell hyperproliferation (Figure 3B), as well as a lack of embryonic blood cells in the vasculature of visceral yolk sacs (VYS) (Figure 3C). Open neural tube, reduced head size and abnormal craniofacial features were also apparent in 0P and 00 embryos. Although we and others have previously reported these phenotypes in non-cultured 0P conceptuses [3], [21], this study represents the first parallel assessment of 0P and 00 embryos derived under the same experimental conditions. Maternal-imprint free embryos were previously reported to gain sporadically methylation at maternal ICRs of the *Peg3* and *Snrpn* loci [18], [22]. We indeed found 25% of 0P and 00 embryos to be normally methylated for one or the other of these loci (data not shown). These two genes also did not reach significant levels of misexpression in our 0P and 00 versus MP comparative microarray analysis, although they tended to be upregulated (data not shown). Remarkably, embryos that had gained normal methylation at *Peg3* or *Snrpn* were not phenotypically distinguishable, in agreement with the fact that these genes are not required for early development and embryonic viability [23]–[25].

Three major conclusions can be drawn from this developmental analysis: *1)* imprint-free 00 and maternal imprint-free 0P embryos cease development at around the 20 somite stage, which corresponds to the time where embryonic development becomes dependent on maternal resource allocation through placental exchanges, *2)* at 8.5dpc, a lack of paternal imprints does not add to the defects seen with a lack of maternal imprints and *3)* simultaneous abolition of maternal and paternal germline imprints does not restore normal development in 00 embryos. To get a more detailed insight into the biological pathways that are dependent upon maternal and paternal imprints, we next functionally dissected the relative changes in the transcriptomes of 00, 0P and MP embryos.

### Maternal ICRs control vital pathways related to the fetal-maternal interface and indirectly impact on genes regulated by paternal ICRs

The transcriptomes of 8.5dpc MP, 0P and 00 embryos were measured using gene expression microarrays. We then determined the genes whose expression levels changed specifically due to a lack of imprints at either maternal or paternal ICRs and identified the gene ontology (GO) categories of biological processes that were most affected by these changes. The minimal phenotypic variation between 00 and 0P embryos assured limited tissue-specific biases.

**Figure 4:**
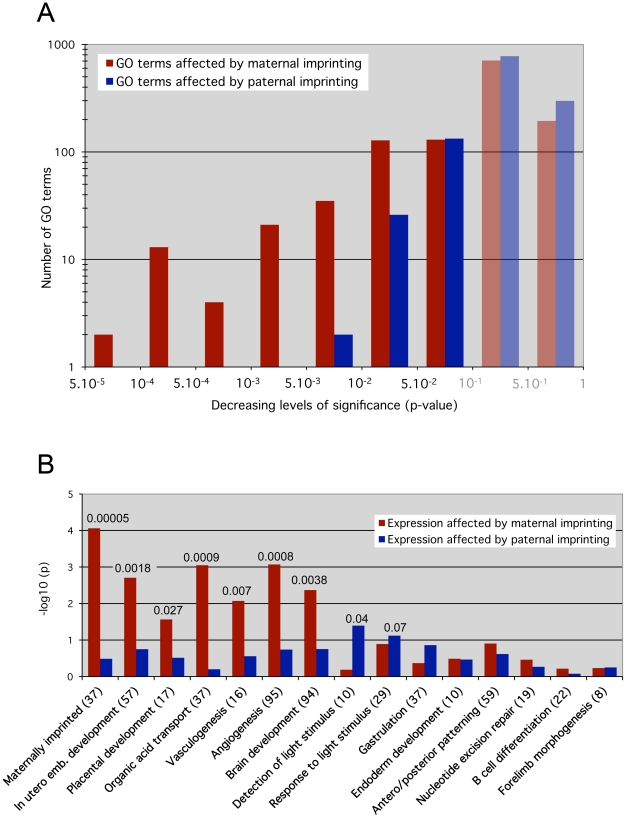
Maternal versus paternal imprint influence on specific molecular functions and developmental processes. Overrepresentation analysis results for Gene Ontology (GO) categories. The analysis was carried out separately and independently for two different scoring schemes. Essentially, the “maternal” scheme assigned a non-zero score if the gene's expression pattern across the MP, 0P and 00 samples was consistent with the gene's expression being affected by maternal but not paternal germline imprints (red bars). Analogously, the “paternal” scoring scheme gave non-zero scores to genes that appeared affected by paternal but not maternal germline imprints (blue bars). *A,* p-value distribution across all GO categories for genes affected by maternal versus paternal imprints. P-values below 0.1 were considered as significant, greater values do not indicate relevant impact (shaded bars). The y-axis shows the –log_10_ value of the GO terms numbers. Note the skewed distribution of paternal-dependent GO terms towards the less significant p-values >0.001. On the contrary, maternal-dependent GO terms are highly clustered on the left towards p <0.001, indicating a stronger biological impact of maternal imprints. *B,* Loss of maternal imprints affects specific early development pathways essential for embryonic viability (*in utero* development, placentation, vasculogenesis, solute transport), while paternal imprints have small effects. The custom Maternally imprinted category served as a control: it was significantly overrepresented (p = 0.00005) under the maternal but not the paternal scoring scheme. The y-axis shows the –log_10_ value of the multiple testing-corrected p-value. The absolute p-values with high confidence scores (<0.1) are reported on top of the corresponding bars. The number of genes present in each biological category is shown in brackets.

The effects of maternal ICRs were assessed by identification of genes that were significantly misexpressed in both 0P and 00 embryos, which both lack maternal imprints compared to MP embryos, but whose expression did not change between 0P and 00 embryos. Analogously, the functional impact of paternal ICRs was determined using genes that were misexpressed in 00 embryos compared to 0P and MP embryos, but did not change between 0P and MP conditions. Under these definitions, the numbers of genes regulated by maternal and paternal ICRs were similar (1695 versus 1581 probe sets, see Table S1). However, a GO overrepresentation analysis revealed that a larger number of biological processes were significantly enriched for genes regulated by maternal versus paternal ICRs: 333 versus 161 GO terms with multiple testing-corrected p<0.1. This difference was even more pronounced for highly significant enriched categories: 75 versus 2 with p<0.01 (Figure 4A). Thus, while maternal and paternal imprints regulate a similar number of genes, the functions of these genes converged onto the same biological processes much more often in the maternal case. In other words, at 8.5dpc, maternal ICRs elicited a much more coordinated effect in terms of gene function.

GO terms include both molecular functions and developmental/cellular processes. The only 2 GO categories that were highly significantly (p<0.01) affected by paternal ICRs were referring to molecular functions: protein ubiquitination (GO:0016567) and protein modification by small protein conjugation (GO:0032446). The developmental processes we identified as significantly affected are in agreement with the activities taking place at 8.5dpc [26]. In particular the expression of genes involved in *in utero* development, placentation, solute transport, vasculogenesis and angiogenesis, key biological processes that are involved in the establishment of the maternal-fetal interface, was highly dependent on maternal imprints (p<0.003) (Figure 4B). Significant upregulation of genes involved in the regulation of angiogenesis (*Serpinf1*, *Adamts1* and *Spint1*) was confirmed in 0P and 00 embryos by real time RT-PCR (data not shown). Global brain development was also preferentially under the control of maternal imprints, although a complementary pattern of parental dependence was observed when specific brain structures were considered (Figure S3). For example, mid- and hindbrain development and light detection were functional categories more significantly affected by paternal than maternal imprints. These observations correlate with previous reports showing that androgenetic PP cells with a pure paternal contribution tend to preferentially colonize hindbrain regions and in particular the pre-optic area in reconstructed chimeric embryos [27]. Further expression analysis of brain development markers may identify differences in neuroectoderm structures between 0P and 00 embryos. Finally, genes involved in gastrulation, antero/posterior patterning, endoderm development, and later developmental processes (B cell development, forelimb morphogenesis) were not significantly affected by maternal or paternal imprints.

The affected biological processes point to defective placentation as the main consequence of a lack of maternal germline imprints and the cause of death of 0P and 00 embryos at mid-gestation. This complements previous studies that have established the importance of genomic imprinting for placentation on a gene-by-gene basis and at later stages of development [28]. Moreover, we show that paternal imprints regulate a large number of transcripts at 8.5dpc, but their cumulative effects do not strongly impact on functions that are vital for the early embryo.

The results of the GO overrepresentation analysis pointed to specific gene families being regulated by imprints of maternal origin. For example, the acid organic transport GO category includes numerous solute-linked carrier (*Slc*) genes. We observed that 100 of 299 of *Slc* genes present on the microarray were either up- or down-regulated in both 00 and 0P embryos. Differential expression of numerous *Slc* genes was previously observed in a microarray study of non-cultured 0P material including pooled embryos and visceral yolk sacs [29]. Slc transporters modulate soluble molecule availability in a variety of physiological contexts, including the regulation of maternal-fetal transfers, and three *Slc* genes are in fact known to be maternally imprinted. To determine whether the abnormally expressed *Slc* genes were directly or indirectly controlled by maternal germline imprints, we analyzed the allelic expression of 25 of these genes that carried informative single nucleotide polymorphisms in reciprocal *Mus musculus* x *Mus musculus castaneus* F1 hybrid crosses. None were subject to parent-specific monoallelic expression in 8.5dpc conceptuses (Table S2). This indicates that a third of all *Slc* genes expressed in early mouse embryos may be downstream targets of maternally imprinted genes.

In summary, these results underline the significant direct and indirect effects that maternal imprints have on the transcriptome of the early embryo, converging towards the vital regulation of genes related to the establishment of the maternal-fetal interface. This bias towards maternal-imprint dependence of the 8.5dpc embryo is likely due to the greater number of maternal ICRs, by impacting on a higher number of imprinted genes at that stage or simply by increasing the chance of at least one of them fulfilling a vital role earlier in development than any one of the paternal ICRs. The reasons for this numerical imbalance are unknown. To better understand the differences in identity and methylation-dependent control of maternal versus paternal ICRs, we analyzed the sequence composition of these sequences in a horizontal (compared to other genomic sequences) and a vertical (during mammalian evolution) perspectives.

### Paternal ICRs differ from related genomic categories in terms of CpG content

Methylated cytosines are susceptible to C to T deamination and the germline methylation status of a sequence is predictive of its likelihood to lose CpG motifs during evolution [16], [17]. Low CpG-content promoters (L), known to be in a methylated state in multiple tissues including the male germline, have lost CpGs at a significantly higher rate than High to Intermediate CpG content promoters (HI) that are constitutively unmethylated [16]. Both maternal and paternal ICRs are methylated in their respective germline. But paternal ICRs are intergenic, and overall, intergenic regions evolve neutrally [30]. In contrast, maternal ICRs coincide with CpG-rich promoters that are under selective pressure for conserving sequence linked to promoter function.

**Figure 5:**
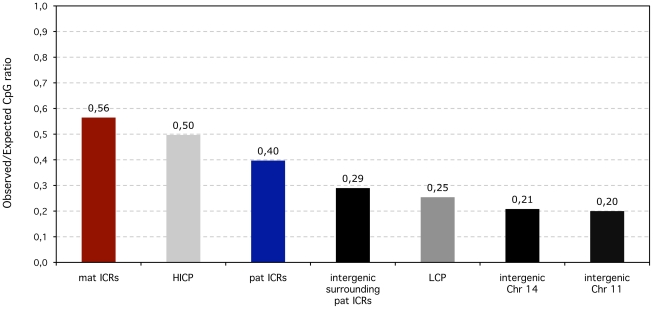
Observed over expected CpG ratios of ICRS compared to non-imprinted promoters and intergenic regions in the human lineage. In terms of CpG content, maternal (mat) ICRs are similar to High to Intermediate CpG content promoters (HICP) (0.56 versus 0.5). Despite being in intergenic regions, the CpG content of paternal (pat) ICRs are higher than for intergenic regions in general (measured along Chromosomes 11 and 14) and also than surrounding intergenic regions. Low (LCP) CpG-content promoters are similar to intergenic regions.

It is therefore unsurprising that paternal ICRs have a significantly smaller observed/expected CpG ratio compared to maternal ones, both in the mouse (0.38 versus 0.49; Fisher's exact test p<10^−7^) and the human genome (0.4 versus 0.56; p<10^−19^ (Figure 5)). We compared the CpG enrichment of ICRs to related genomic sequences, and in particular to HI and L promoters and to intergenic regions. We found that all but one maternal ICRs meet the criteria of HI promoters, and were even more CpG-rich than the average non-imprinted HI promoters (0.56 versus 0.5) (Figure 5). Unexpectedly, paternal ICRs have a different nucleotide composition than their related sequence category, being significantly more enriched in CpGs than random intergenic sites, including the ones that constitute their immediate surrounding environment (0.4 versus 0.29). This relative enrichment is also maintained when compared to Low CpG content promoters (Figure 5).

Hence, despite being methylated in the female germline, maternal ICRs have the same CpG content as constitutively methylation-free HI promoters. In contrast, paternal ICRs have an excess of CpG motifs compared to any non-imprinted genomic sequence- intergenic or promoter-associated- that exists in a methylated state in the male germline, leaving up the possibility that paternal ICRs may have maintained or gained CpGs. Intergenic versus promoter position is therefore not sufficient to explain the discrepancy between paternal and maternal ICRs. We previously suggested that the lower CpG content of paternal ICRs may reflect their longer exposure to methylation-induced mutagenesis in the male germline, compared to maternal ICRs that have a very brief existence in a methylated state during oogenesis [5], [6]. This hypothesis was however never empirically tested. To shed light onto the mechanisms that have shaped the unique CpG content of maternal versus paternal ICRs during mammalian evolution, we thus adopted a comparative genomics approach that is capable of inferring rates of dinucleotide substitutions from multiple sequence alignment data for species whose phylogeny is known [30]. This approach was previously used to compare the rates of CpG loss between HI and L promoters [16]. We included these two sequence categories in our analysis predominantly as internal controls to assure that we could reproduce these results. However, since all maternal ICRs are HI promoters in term of CpG content, the inclusion of non-imprinted HI promoters also enabled us to investigate how imprinting of a CpG-rich promoter affects the evolution of CpG methylation targets.

### Paternal ICRs have lost CpGs, while maternal ICRs gain CpGs, during mammalian evolution

We inferred rates of CpG-loss and -gain for 2 paternal and 13 maternal ICRs with strong evidence for sequence, differential methylation (imprinting) and functional conservation between human and mouse (Table S3). We then assumed ICR conservation in all extant species that descended from the last common ancestor of human and mouse and retrieved multiple alignment data of the corresponding human genomic sequences with 15 other euarchontoglire species (8 primates, treeshrew, 4 rodents, 2 lagomorphs) to form the basis for the inference of evolutionary models using Ambiore [30]. The inclusion of the sequence data for euarchontoglire species other than human and mouse was necessary to obtain sufficient statistical power, especially in the case of paternal ICRs.

**Figure 6:**
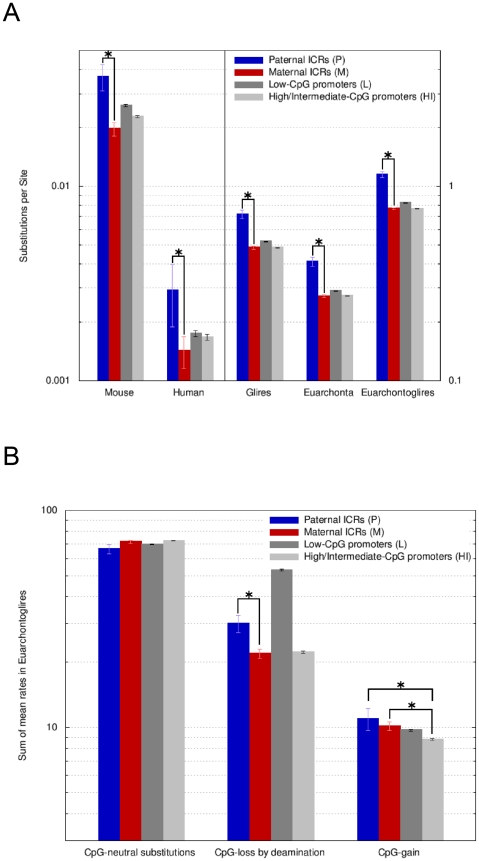
Paternal ICRs have evolved faster than maternal ICRs and have endured a higher rate of CpG loss by deamination. *A,* Overall amount of nucleotide changes at paternal and maternal ICRs. Amount of change is expressed along the y-axis (log_10_ scale) as the sum of the Ambiore-estimated branch lengths for singleton branches (human, mouse), for the respective sub-tree (euarchonta, glires) or for the entire tree (euarchontoglires). Sequence categories are: P =  paternal ICRs, M =  maternal ICRs, L =  Low CpG-content promoters, HI  =  High and Intermediate CpG-content promoters. Error bars are 95% confidence intervals. Significant (non-overlapping 95% confidence intervals) changes of interest are labelled with asterisks. Paternal ICRs present with the most evolutionary change, compared to all other sequence types and to maternal ICRs in particular. The values for human and mouse are two orders of magnitude lower than for euarchontoglires, euarchonta and glires. Hence, the use of two y-axis scales (left versus right). *B*, Rates of substitutions occurring at CpG dinucleotides in euarchontoglires. The estimated substitution rates are relative to each category's overall rate of evolution, e.g., the fact that paternal ICRs are fast evolving intergenic regions, while all other categories are promoter-associated, has been normalized for. Nevertheless, the rate of CpG-loss by deamination at paternal ICRs is higher than for maternal ICRs. Maternal ICRs loose CpGs at the same pace as HI promoters but gain CpGs at a faster rate.

An Ambiore-inferred evolutionary model consists of estimates of absolute amounts of sequence change (branch lengths of the given phylogenetic tree on a scale of substitutions per site) and a rate for each possible context-dependent nucleotide substitution. The substitution rates reported by Ambiore are independent of the overall different speeds with which intergenic and promoter regions evolved, that is in our case, within a sequence category, each rate expresses the frequency of CpG substitution relative to all substitutions that occurred (Dick Hwang; personal communication). That enables the direct comparison of CpG-loss and -gain rates between sequence categories like maternal and paternal ICRs, despite the latter having experienced many more substitution events than any of the three promoter categories, which is consistent with paternal ICRs being intergenic (Figure 6A).

Despite this implicit normalization, we found that the rate of CpG loss was considerably (1.5-fold) and significantly greater for paternal ICRs than for maternal ICRs (Figure 6B). CpG loss was predominantly due to deamination, with the contribution of other substitution types being negligible (data not shown). Maternal ICRs showed a similar rate of CpG loss than non-imprinted HI promoters. On the other hand, the rate of CpG loss at paternal ICRs was much smaller than at L promoters, despite the overall faster evolution of intergenic paternal ICRs and the constrained evolution of L promoters linked to the pressure to maintain transcription-initiation sites (Figure 6A). Our results recapitulate and extend the previously published observation that L promoters exhibit a high rate of CpG loss relative to HI promoters [16], and are consistent with our observation that paternal ICRs have nowadays a greater CpG content than L promoters in the human lineage (Figure 5). In terms of CpG gain, paternal and maternal ICRs were indistinguishable (Figure 6B), both showing a slightly yet significantly greater rate of CpG gain than non-imprinted HI promoters. These findings were confirmed when the data were split into the euarchonta and glire clades and reanalyzed, and also when we used PhyloFit [31] instead of Ambiore for evolutionary model inference (Figures S4 and S5). However, overall, paternal ICRs still lose CpGs relative to HI promoters since the difference in the CpG loss rate between these two categories by far exceeds the difference in the CpG gain rate. For maternal ICRs, the loss rate is equal to HI promoters, so that the higher rate of CpG gain translates into an actual gain of CpGs relative to HI promoters over time.

Since substitution rates are independent of the overall speed with which a sequence category evolved (see above), the higher rate of CpG loss by deamination in paternal versus maternal ICRs cannot be attributed to the intergenic location of paternal ICRs. On the other hand, CpG loss in paternal ICRs has been slower than in L promoters that are similarly methylated in the male germline, suggesting that there has been positive selection pressure to maintain the CpGs of paternal ICRs. However, this positive pressure appears to have been insufficient to completely neutralize the difference in deamination rates between maternal and paternal ICRs, consistent with higher mutational pressure due to deamination in the paternal compared to the maternal germline. Finally, the higher rate of CpG gain in maternal ICRs relative to non-imprinted HI promoters indicates that the accumulation of methylation targets is subject to positive selection at maternal ICRs.

## Discussion

Our investigation of the transcriptome-wide effects of maternal and paternal ICRs, the regulatory sequences that govern genomic imprinting in mammals, provides the first unbiased view of their respective functional significance for the early embryo at the time of establishment of the fetal-maternal interface (8.5dpc). A previous genome-wide study was aimed at the identification of gene networks that specifically depend on paternal imprints at later stages of development (12.5 and 15.5dpc) and did not include a systematic characterization of the involved biological processes [20]. Our work was motivated by previous observations in mouse models of global imprinting deficiency that pointed towards an earlier requirement of maternal versus paternal ICRs for mammalian development. In particular, complete maternal imprint deficiency arrests development at 9.5dpc [3], while a lack of all paternal imprints does not affect embryonic viability before 13.5dpc [12].

We found that at 8.5dpc, maternal and paternal ICRs affected the expression of a similar number of genes, but when the genes were assigned functional categories according to the Gene Ontology (GO terms), a pronounced asymmetry became apparent. Only genes affected by maternal ICRs were significantly overrepresented in functional categories related to placentation and mother-to-embryo exchanges. In contrast, the effect of paternal ICRs on the transcriptome was unfocused, significantly affecting relatively few functional categories overall and none related to the fetal-maternal interface. In addition, a lack of maternal imprints had a significant impact on the expression of paternally imprinted genes, presumably via the *Zac1*-centered gene network [19], while a lack of paternal imprints did not significantly alter the expression of maternally imprinted genes. We propose that this functional dominance of maternal ICRs at 8.5dpc explains why maternal-imprint free embryos (0P and 00) never reach later developmental stages (13.5dpc and beyond) when paternal imprints become crucial for development. The sporadic reacquisition of *Peg3* and *Snrpn* methylation in some embryos does not compromise our conclusion about this prominent role and may even have led to an underestimation of the maternal impact, provided that these genes have any significant role at 8.5dpc, a feature that is not supported by our phenotypic analysis and by former gene inactivation studies [23]–[25].

Individual deletions of imprinted genes, although resulting in a different outcome compared to the abolition of imprints, are often embryonic lethal and have shaped the notion of a strong functional association between genomic imprinting and the placenta. For example, the inactivation of the maternally imprinted genes *Peg10* or *Ascl2* leads to early embryonic lethality due to placental defects [32], [33]. However, among the three paternally imprinted loci, only the *Dlk1/Gtl2* gene cluster exerts a vital effect on placentation at 16.5dpc [34], [35], while misregulation of the two others does not prevent full term *in utero* development [36], [37]. Our findings on the global functional impact of all paternal versus all maternal imprints at 8.5dpc are consistent with these previous observations and provide additional evidence for a strong link between placental function and imprinting, a relationship in which maternal imprints appear to dominate in the early stages.

The functional link and the temporal coincidence of the evolutionary origins of the placenta and genomic imprinting suggest that placenta and genomic imprinting co-evolved [13], [28]. Specifically, one can consider the evolution of the placenta to have presented a new gene regulatory challenge for eutherian mammals that may have been met by the evolution of imprinting. Selection pressure originating with the placenta to tightly regulate the expression of key genes involved in placental function could explain the evolution of the imprinting mechanism and subsequent accumulation of imprinted loci during eutherian evolution. But it does not explain the numerical dominance of maternal ICRs in extant eutherian genomes. We have previously proposed [5], [6] and here, have provided evidence that differential mutational pressure on methylated sequences between the two parental germlines can explain the preferential accumulation of maternal ICRs during evolution.

In the male germline, methylation patterns are established prior to birth and can last for the entire lifespan of an individual due to the self-renewal activity of spermatogonial stem cells. In humans, this represents 65 years on average and several hundred cell divisions. In the female germline on the other hand, methylation patterns are maintained for only a few days before ovulation and in the absence of DNA replication. Considering that the methylation of cytosines significantly increases the rate of deamination, that is, C to T transition mutations [14], [15], [17], the rate of CpG loss due to deamination is expected to be higher in paternal versus maternal ICRs. Here, we have demonstrated that this has indeed been the case during eutherian evolution, at least since the divergence of glires and euarchonta. Maternal ICRs, all of which coincide with CpG-rich promoters, have experienced a similar rate of CpG loss due to deamination compared to non-imprinted CpG-rich promoters that are constitutively unmethylated. This is consistent with maternal ICRs being only briefly and thus insignificantly exposed to the mutagenic effect of methylation during their passage through the female germline.

We also found evidence for selection pressure favoring the maintenance of methylation targets in paternal ICRs in comparison to other sequences that are methylated in the male germline. Paternal ICRs constitute some local enrichment in CpG sites over the globally CpG-depleted intergenic landscape. They have also a higher CpG density than L promoters in the human genome, which we show, results from a higher resistance to CpG loss during mammalian evolution. This is consistent with the functional significance of DNA methylation at ICRs in controlling gene expression, while the methylation state of L promoters does not affect the transcription level of associated genes [16]. Although the underlying mechanisms have not been identified, protection against CpG loss at paternal ICRs could result from increased efficiency of T/G mismatch repair, or from reduced deamination frequency of methylated cytosines, entailed for example by local DNA structure. In this regard, replication and transcription generate ssDNA, in which cytosines residues deaminate much more rapidly than in dsDNA [38]: relative localization of replication origins or transcription start sites in intergenic paternal ICRs versus L promoters may result in different CpG loss rate between these two sequence categories. Independently of the parental origin, paternal and maternal ICRs also accumulate new CpG sites during evolution, gaining more CpGs than non-imprinted HI promoters. Imprinted chromosomal regions have unusually high rates of meiotic recombination compared to the rest of the human genome [39], [40]. This property could drive the accumulation of CpG sites at ICRs during meiotic repair through biased gene conversion, a process that favors the fixation of AT to GC mutations [41]. Whichever process acts to conserve or create CpG sites in ICRs versus the rest of the genome, it appears to have been insufficient in the long term to counteract the hyper-mutagenic environment of the male germline. Only three functional paternal ICRs have been identified in mouse and genetic manipulation of paternally imprinted expression suggests that this may represent the total number of all developmentally important ICRs controlled by paternal methylation [20]. A fourth intergenic locus undergoing paternal-specific methylation has been recently characterized, but its function as an ICR has not been ascertained yet [9]. It nonetheless has likely been exposed to the evolutionary forces that we describe here, with an obs/exp CpG ratio within the range we defined for paternal ICRs (0.34).

Taken together, our results suggest that the functional dominance of maternal ICRs during early embryonic development is the consequence of two orthogonal evolutionary forces: *1)* selection pressure to tightly regulate the expression of genes affecting the fetal-maternal interface once the placenta had evolved, increasing the number of imprinted loci *per se* and the number of CpG methylation targets, and *2)* simultaneous pressure to avoid the deamination-prone environment of the paternal germline, favoring the evolution of maternal ICRs. The resulting numerical dominance of maternal ICRs implies a greater chance of some maternal ICRs to fulfill a vital role earlier in development than any one of the paternal ICRs, explaining the earlier lethality of maternal imprint deficiency and their functional dominance over the fetal-maternal interface at the time of its establishment. These two forces may have been aided by an intrinsic ability of the female germline to methylate CpG-rich regions. Indeed, we previously showed that de *novo* insertions of CpG-dense sequences are naturally targeted by methylation in the oocyte, provided that the insertion happened in an active transcription unit [42]. Mechanistic reasons for this association were more recently provided, by demonstrating that maternal ICRs need to be traversed by upstream transcripts to be methylated in the oocyte [43]. The exceptionally high transcriptional activity of the growing oocyte related to the necessity to establish a maternal store [44] may therefore have led to a propensity for the oocyte to methylate genes associated with CpG-rich promoters. Oocyte-methylation is then maintained after fertilization at a few loci, for the purpose of controlling expression levels of developmentally important genes and notably related to the vital transition step towards maternal-fetal exchanges.

## Materials and Methods

### Sequence data

The positions in the March 2006 human genome build (hg18) of 13 maternal and 2 paternal germline ICRs that are definitively (*KCNQ1OT1, ZAC1, MEST, ZIM2, GNAS-EXON1A, SNURF/SNRPN, PEG10, GRB10, H19/IGF2* ICR, *GTL2/DLK1* IG-DMR*)* or likely (*NNAT, INPP5F_V2, NAP1L5, MCTS2, PEG13*) conserved between human and mouse were determined from published methylation data (Table S1). The positions of 3,530 validated Low (L) CpG-content promoters and 10,872 High to Intermediate (HI) CpG-content promoters were extracted from [16]. The 12 maternal ICRs that fell into the HI category were excluded from the HI category. Definition of genomic intervals and euarchontoglire species used to retrieve multiple alignment data are presented in Text S1.

### Evolutionary model estimation

Strand-symmetric context-dependent substitution rates and branch lengths were estimated using Ambiore and PhyloFit [30], [31]. The topology of the phylogenetic tree for euarchontoglires was taken from the 44-species UCSC conservation track of the human genome [45]. Details of the methodology are provided in Text S1.

### Generation and epigenotype confirmation of MP, 0P, and 00 embryos

Details of the procedure are provided as supplemental information. Conceptuses were dissected at 8.5, 9.5 and 10.5dpc (relative to the foster mother) and VYS were genotyped: MP were *Dnmt3L+/+*, 0P *Dnmt3L−/+*, and 00 *Dnmt3L−/−*. Epigenotypes were confirmed by assessing the methylation status of the *H19* and *Kcnq1ot1* ICRs by bisulfite sequencing, before inclusion on the microarray.

### Microarray creation and analysis

All samples were assayed using Affymetrix Mouse MOE430v2 expression microarrays. Four 8.5dpc embryos with confirmed genotype and epigenotype were pooled per category (MP, 0P and 00) to account for individual biological diversity. Five to seven µg of total RNA was used per sample as input. Probe level summarization was performed using the Affymetrix GCOS/MAS5 (target value of 500; otherwise default parameters) and GC-RMA (ArrayAssist implementation; default parameters) algorithms [46]. Further details are provided in [29].

### Gene ontology (GO) analysis

Only non-control probe sets whose target sequences could be BLAT-aligned [47] uniquely and with high identity (80%) to a single location within the mouse genome (NCBI build 36) were considered. Probe sets that did not detect expression in either MP, 0P or 00 (GCOS/MAS5-computed detection p-value always >0.06) were excluded. To eliminate any sex-specific effects secondary to the obligate female gender of parthenogenetic 00 embryos, probe sets mapping to Chr Y or the *Xist* locus on Chr X were not included in the analysis.

Sets of genes specifically affected by the absence of maternal and paternal methylation imprints were determined as explained in Text S1. On the basis of the respective list of scored probe sets, a GO category overrepresentation analysis was carried out using ErmineJ [48] (v2.1.13) with the GO term database and Affymetrix MOE430v2 probe set annotation (Apr 13, 2007). The score threshold was set to 0.01 so that relatively small changes in expression were considered relevant.

## Supporting Information

Figure S1 Confirmation of expected parental direction of imprinted gene expression in 8.5dpc 0P and 00 relative to MP embryos. *A,* RNA blot hybridization analysis of imprinted gene expression. The *H19* and *Igf2* genes regulated by the same paternal ICR are specifically misexepressed in 00 embryos. The *Igf2r* and *Cdkn1c* regulated by 2 independent maternal ICRs are downregulated in both 0P and 00 embryos. *B,* Real-time PCR was used to determine the expression profile of four inversely regulated pairs of clustered genes: *Kcnq1ot1*-*Cdkn1c* and *Airn*-*Igf2r* genes regulated by maternally methylated ICRs (upper part), and *Gtl2*-*Dlk1* and *H19*-*Igf2* genes regulated by paternally methylated ICRs (lower part). As expected, both 0P and 00 embryos showed an increased expression of the maternally repressed *Kcnq1ot1* and *Airn* non-coding RNAs and a subsequent downregulation of *Cdkn1c* and *Igf2r* transcripts. Only 00 embryos showed a significant upregulation of the paternally repressed *Gtl2* and *H19* genes and a subsequent downregulation of *Dlk1* and *Igf2* genes. Values were normalized to *beta-actin* expression level and were calibrated to the expression level in MP embryos. The number of analyzed embryos per category is reported into brackets. Results are represented as mean fold differences versus MP embryos ±SD.
(0.97 MB TIF)

Figure S2 Imprint-free 00 embryos are arrested at 8.5dpc. 00 embryos are similar in development to 8.5dpc embryos at 9.5 (A) and 10.5dpc (B) compared to MP embryos transferred in the same uterine horns.
(2.22 MB TIF)

Figure S3 Influence of maternal and paternal imprints on specific brain structure development revealed by gene ontology analysis of MP, 00 and 0P embryo transcription profiles. While maternal imprints dominantly affect genes important for global brain development at 8.5dpc (p<0.003), their influence is more pronounced in forebrain structures while mid- and hindbrain regions are rather under the influence of genes regulated by paternal imprints. Report to Figure 4 for graph legend.
(0.17 MB TIF)

Figure S4 Rates of substitution occurring at CpG dinucleotides, analogous to Figure 6B, except that values were split into the euarchonta portion in *A* and the glire portion in *B*. The overall profiles of mean rates across sequence categories is largely unchanged compared to results obtained with all euarchontoglire species, with paternal ICRs exhibiting a higher CpG deamination rate than maternal ICRs.
(0.60 MB TIF)

Figure S5 Estimation of substitution rates using PhyloFit with a symmetric, non-reversible, trinucleotide context-dependent substitution model (U3S). Results were qualitatively and quantitatively similar to Ambiore. *A*, Increased overall rate of substitution at any nucleotide for paternal ICRs compared to maternal ICRs and to all other promoter-associated sequence categories (as in Figure 6A). *B*, Increased CpG loss by deamination at paternal ICRs compared to maternal ICRs and increased CpG gain of maternal ICRs compared to non-imprinted HI promoters. Note that PhyloFit does not estimate confidence intervals.
(0.62 MB TIF)

Table S1 A total of 1695 probe sets detected a significant change in expression in response to a lack of maternal but not paternal imprints (column M). A lack of paternal but not maternal imprints resulted in 1582 probe sets signaling a significant change in expression (column P). See GO Analysis in Materials and Methods for the complete definition of the M and P probe set categories. The table shows a break-down of these total numbers into categories according to the minimally detected fold-change (log2-ratio). In the maternal case for example, 470 probes sets detected a decrease of expression in the 0P and 00 samples to 80% (-0.322 log2-ratio) or less relative to the MP sample.
(0.04 MB DOC)

Table S2 List of 25 *Slc* genes significantly misexpressed by the lack of maternal imprints but not directly imprinted. These genes were confirmed to be biallelically expressed in F1 hybrid 8.5dpc embryos.
(0.05 MB DOC)

Table S3 ICRs and associated promoter/feature positions for human and mouse.
(0.08 MB XLS)

Text S1 Supporting Material and Methods
(0.10 MB DOC)
