# Supplementary figures and images for: The Parental Non-Equivalence of Imprinting Control Regions during Mammalian Development and Evolution
Source: PLoS Genet. 2010 Nov 18;6(11):e1001214. doi: 10.1371/journal.pgen.1001214 (PMC2987832; doi:10.1371/journal.pgen.1001214)

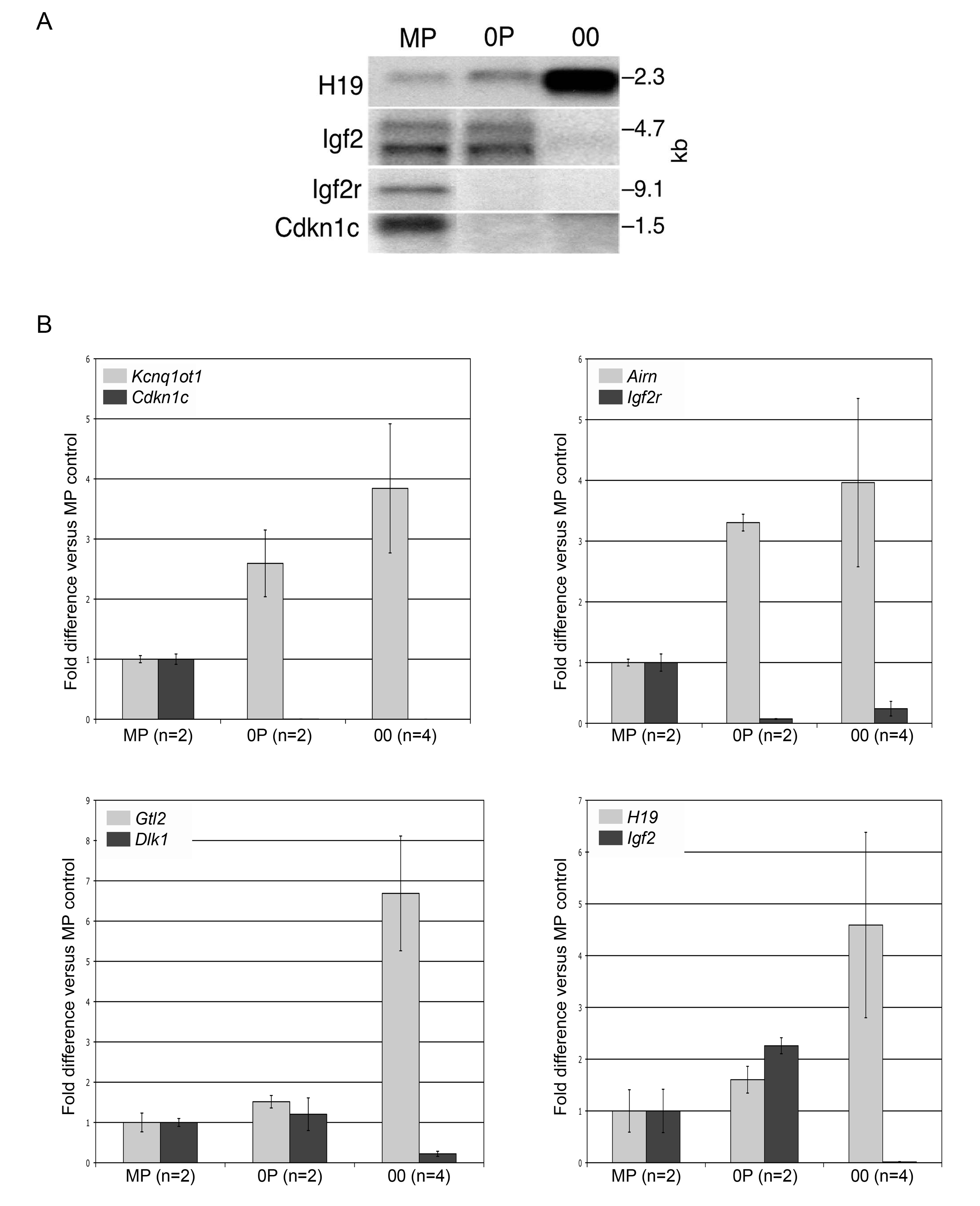

Supplement: Figure S1 — Confirmation of expected parental direction of imprinted gene expression in 8.5dpc 0P and 00 relative to MP embryos. A, RNA blot hybridization analysis of imprinted gene expression. The H19 and Igf2 genes regulated by the same paternal ICR are specifically misexepressed in 00 embryos. The Igf2r and Cdkn1c regulated by 2 independent maternal ICRs are downregulated in both 0P and 00 embryos. B, Real-time PCR was used to determine the expression profile of four inversely regulated pairs of clustered genes: Kcnq1ot1-Cdkn1c and Airn-Igf2r genes regulated by maternally methylated ICRs (upper part), and Gtl2-Dlk1 and H19-Igf2 genes regulated by paternally methylated ICRs (lower part). As expected, both 0P and 00 embryos showed an increased expression of the maternally repressed Kcnq1ot1 and Airn non-coding RNAs and a subsequent downregulation of Cdkn1c and Igf2r transcripts. Only 00 embryos showed a significant upregulation of the paternally repressed Gtl2 and H19 genes and a subsequent downregulation of Dlk1 and Igf2 genes. Values were normalized to beta-actin expression level and were calibrated to the expression level in MP embryos. The number of analyzed embryos per category is reported into brackets. Results are represented as mean fold differences versus MP embryos ±SD. (0.97 MB TIF) [file pgen.1001214.s001.tif]

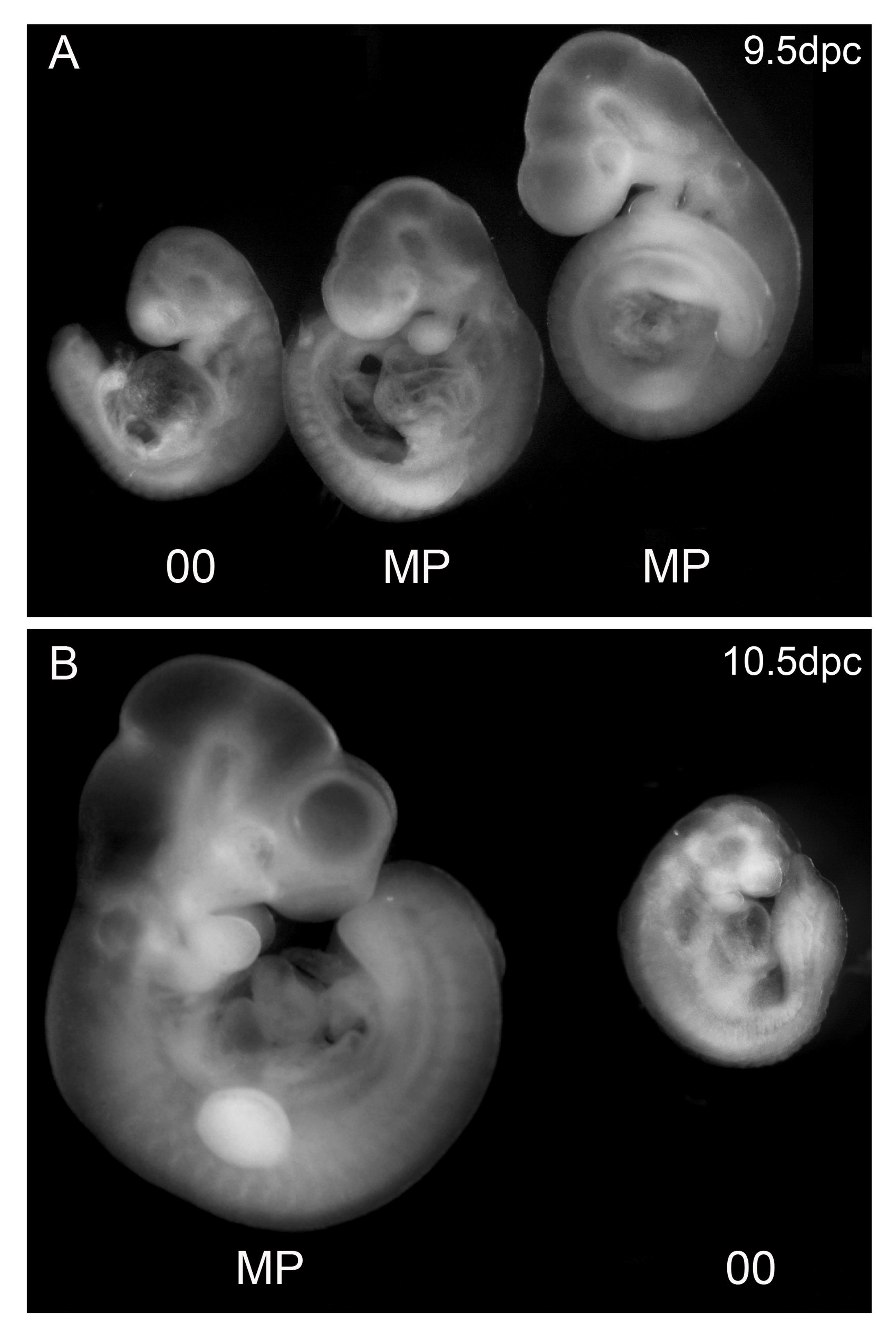

Supplement: Figure S2 — Imprint-free 00 embryos are arrested at 8.5dpc. 00 embryos are similar in development to 8.5dpc embryos at 9.5 (A) and 10.5dpc (B) compared to MP embryos transferred in the same uterine horns. (2.22 MB TIF) [file pgen.1001214.s002.tif]

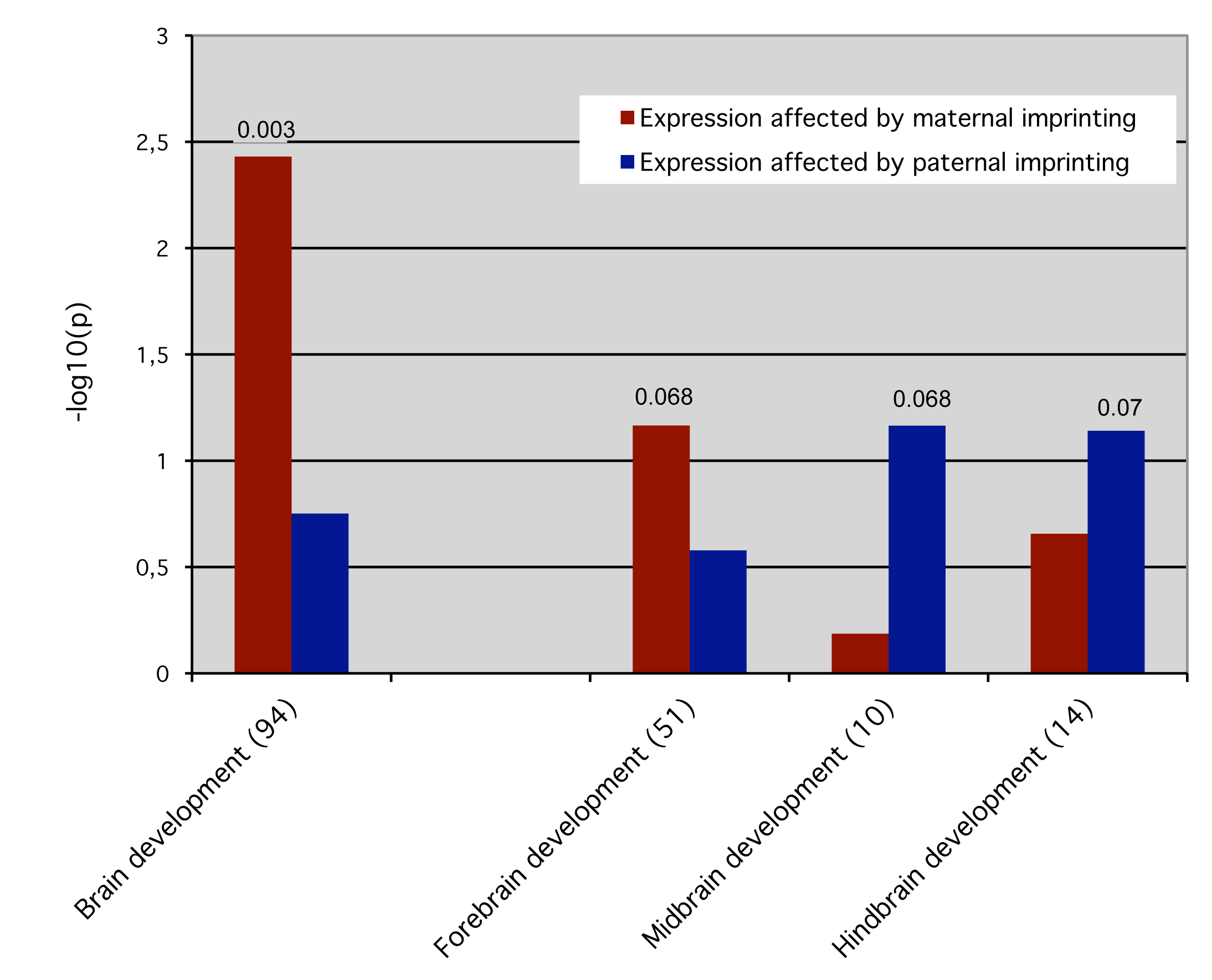

Supplement: Figure S3 — Influence of maternal and paternal imprints on specific brain structure development revealed by gene ontology analysis of MP, 00 and 0P embryo transcription profiles. While maternal imprints dominantly affect genes important for global brain development at 8.5dpc (p<0.003), their influence is more pronounced in forebrain structures while mid- and hindbrain regions are rather under the influence of genes regulated by paternal imprints. Report to Figure 4 for graph legend. (0.17 MB TIF) [file pgen.1001214.s003.tif]

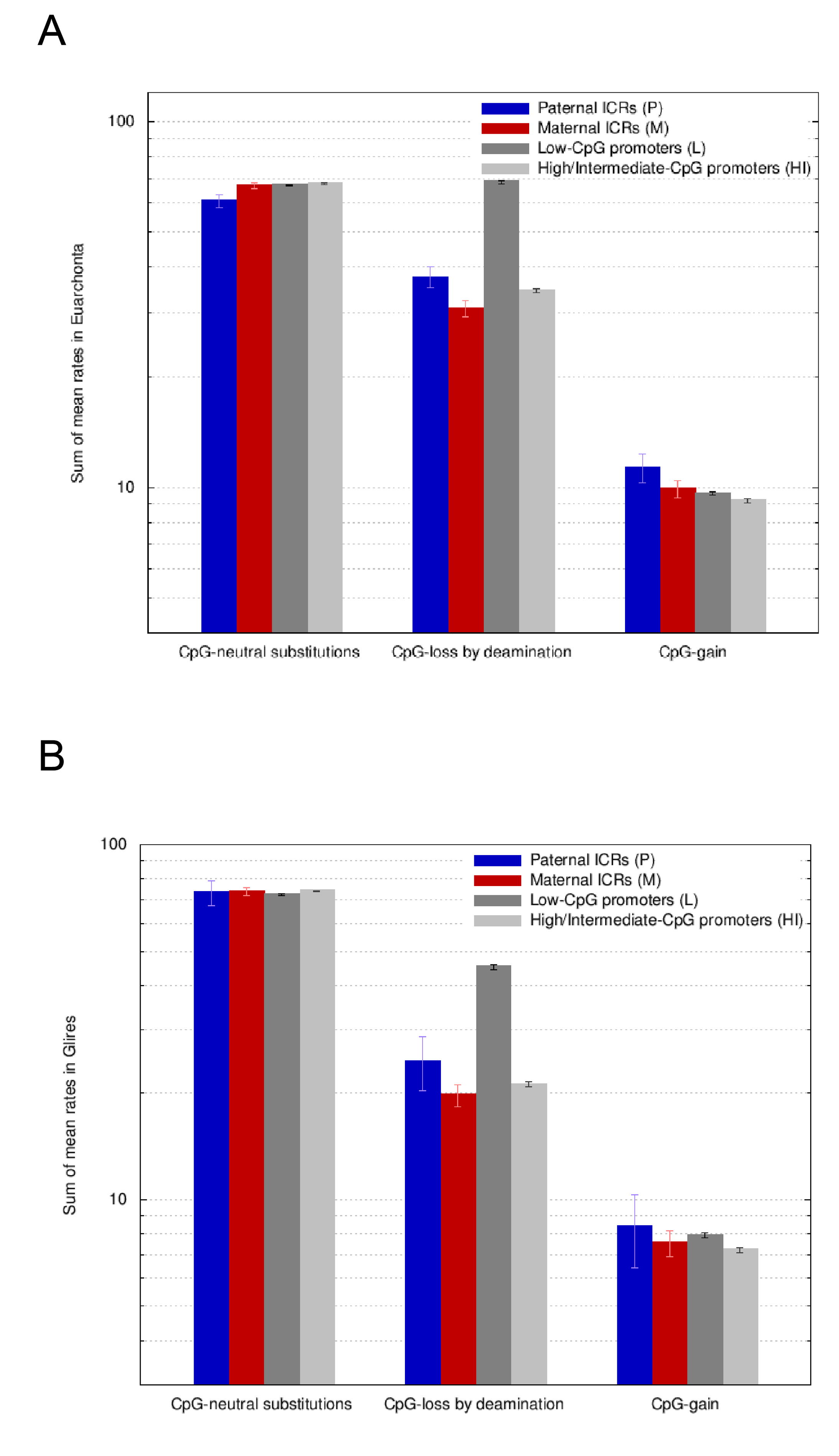

Supplement: Figure S4 — Rates of substitution occurring at CpG dinucleotides, analogous to Figure 6B, except that values were split into the euarchonta portion in A and the glire portion in B. The overall profiles of mean rates across sequence categories is largely unchanged compared to results obtained with all euarchontoglire species, with paternal ICRs exhibiting a higher CpG deamination rate than maternal ICRs. (0.60 MB TIF) [file pgen.1001214.s004.tif]

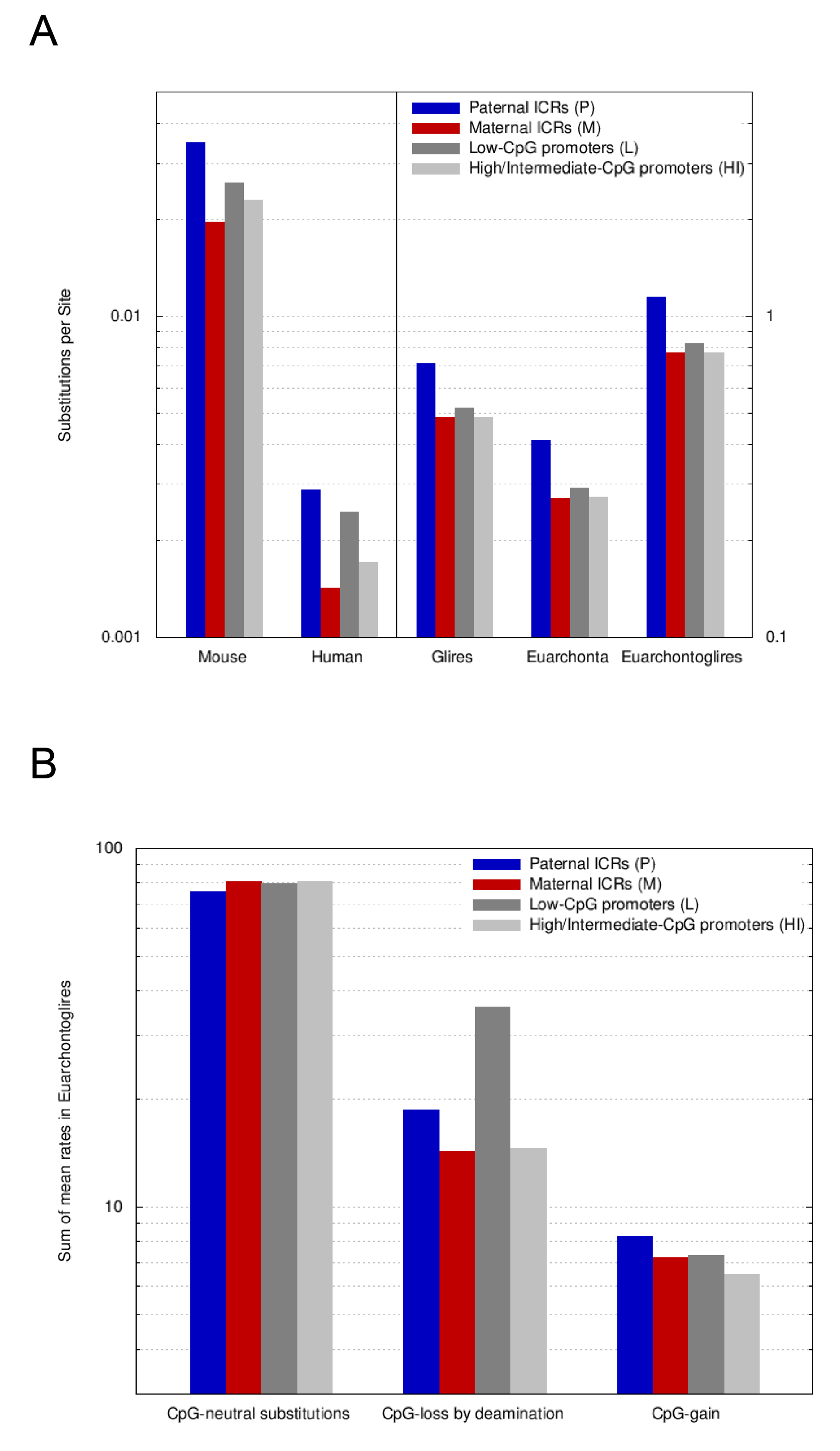

Supplement: Figure S5 — Estimation of substitution rates using PhyloFit with a symmetric, non-reversible, trinucleotide context-dependent substitution model (U3S). Results were qualitatively and quantitatively similar to Ambiore. A, Increased overall rate of substitution at any nucleotide for paternal ICRs compared to maternal ICRs and to all other promoter-associated sequence categories (as in Figure 6A). B, Increased CpG loss by deamination at paternal ICRs compared to maternal ICRs and increased CpG gain of maternal ICRs compared to non-imprinted HI promoters. Note that PhyloFit does not estimate confidence intervals. (0.62 MB TIF) [file pgen.1001214.s005.tif]
