# Supplementary material for: The Parental Non-Equivalence of Imprinting Control Regions during Mammalian Development and Evolution
Source: PLoS Genet. 2010 Nov 18;6(11):e1001214. doi: 10.1371/journal.pgen.1001214 (PMC2987832; doi:10.1371/journal.pgen.1001214)
Supplement: Table S1 — A total of 1695 probe sets detected a significant change in expression in response to a lack of maternal but not paternal imprints (column M). A lack of paternal but not maternal imprints resulted in 1582 probe sets signaling a significant change in expression (column P). See GO Analysis in Materials and Methods for the complete definition of the M and P probe set categories. The table shows a break-down of these total numbers into categories according to the minimally detected fold-change (log2-ratio). In the maternal case for example, 470 probes sets detected a decrease of expression in the 0P and 00 samples to 80% (-0.322 log2-ratio) or less relative to the MP sample. (0.04 MB DOC) [file pgen.1001214.s006.doc]

Schulz_ Table S1

|  | M | P |
| --- | --- | --- |
| Total number of probe sets | 1695 | 1582 |
| log2-ratio (fold change) |  |  |
| -3 (1/8) | 3 | 20 |
| -2 (1/4) | 12 | 76 |
| -1.585 (1/3) | 26 | 145 |
| -1 (1/2) | 75 | 394 |
| -0.585 (2/3) | 308 | 838 |
| -0.322 (4/5) | 470 | 1031 |
| 0.322 (5/4) | 1012 | 492 |
| 0.585 (3/2) | 707 | 372 |
| 1 (2) | 202 | 40 |
| 1.585 (3) | 79 | 11 |
| 2 (4) | 30 | 4 |
| 3 (8) | 11 | 1 |
