# Supplementary material for: The Parental Non-Equivalence of Imprinting Control Regions during Mammalian Development and Evolution
Source: PLoS Genet. 2010 Nov 18;6(11):e1001214. doi: 10.1371/journal.pgen.1001214 (PMC2987832; doi:10.1371/journal.pgen.1001214)
Supplement: Table S2 — List of 25 Slc genes significantly misexpressed by the lack of maternal imprints but not directly imprinted. These genes were confirmed to be biallelically expressed in F1 hybrid 8.5dpc embryos. (0.05 MB DOC) [file pgen.1001214.s007.doc]

Schulz_ Table S2

| probeSet | symb_Affy |
| --- | --- |
| 1433734_at | Slc13a4 |
| 1415802_at | Slc16a1 |
| 1453675_at | Slc16a10 |
| 1454104_a_at | Slc16a9 |
| 1441315_s_at | Slc19a2 |
| 1445589_at | Slc23a2 |
| 1416955_at | Slc25a10 |
| 1444489_at | Slc25a12 |
| 1452653_at | Slc25a22 |
| 1416275_at | Slc26a6 |
| 1424441_at | Slc27a4 |
| 1450639_at | Slc28a2 |
| 1451782_a_at | Slc29a1 |
| 1449067_at | Slc2a2 |
| 1455898_x_at | Slc2a3 |
| 1436164_at | Slc30a1 |
| 1418843_at | Slc30a4 |
| 1455285_at | Slc31a1 |
| 1429649_at | Slc35a3 |
| 1428793_at | Slc36a1 |
| 1425364_a_at | Slc3a2 |
| 1434502_x_at | Slc4a1 |
| 1417150_at | Slc6a4 |
| 1450982_at | Slc9a3r1 |
| 1435009_at | Slc9a6 |
