# Supplementary material for: The Parental Non-Equivalence of Imprinting Control Regions during Mammalian Development and Evolution
Source: PLoS Genet. 2010 Nov 18;6(11):e1001214. doi: 10.1371/journal.pgen.1001214 (PMC2987832; doi:10.1371/journal.pgen.1001214)
Supplement: Text S1 — Supporting Material and Methods (0.10 MB DOC) [file pgen.1001214.s009.doc]

**SUPPORTING INFORMATION**

**MATERIAL and METHODS**

**Alignment data pre-processing**

Anchor points in the human genome were defined for promoter-associated sequences and intergenic regions as follow. For promoter-associated sequences (M, L and HI), the transcription start site (TSS) coordinates served as the anchor point for a 6kb region extending 1.2kb upstream and 4.8kb downstream of the TSS. Intergenic regions were defined as regions in between UCSC genes [http://www.ncbi.nlm.nih.gov/pubmed/16500937] limited to the parts without an overlap with the 6kbp windows for the other sequence categories. Intergenic regions surrounding paternal ICRs were defined to be within 300kbp. Telomeric and centromeric regions were excluded from the set of intergenic regions on Chrs 11 and 14. For intergenic paternal ICRs (P), the midpoint of the union of the regions found to be methylated in the paternal germline was used as the anchor and mid-point of a 6kb region. For each 6kb region, multiple alignment data for all euarchontoglire species present in the 44-species UCSC conservation track of the human genome were retrieved from the UCSC genome browser using Galaxy (<http://galaxy.psu.edu/>). The species are: chimp (panTro2), gorilla (gorGor1), orangutan (ponAbe2), rhesus (rheMac2), marmoset (calJac1), tarsier (tarSyr1), mouse lemur (micMur1), bushbaby (otoGar1), treeshrew (tupBel1), mouse (mm9), rat (rn4), kangaroo rat (dipOrd1), guinea pig (cavPor3), squirrel (speTri1), rabbit (oryCun1), pika (ochPri1).

To increase confidence in the multiple alignment (MA) data and to enable the determination of the direction of substitution events by parsimony, each of the retrieved MAs was trimmed so that only those alignment blocks remained that were gap-free for

- at least one great ape (chimp, gorilla, orangutan) and
- at least one species out of rhesus, tarsier, marmoset and treeshrew, and
- at least two euarchonta that are not great apes and
- mouse and
- at least one other rodent and
- two other glires.

However, gaps of less than 10bp were tolerated. The remaining blocks of each MA were concatenated, joined with triple-columns of N to avoid spurious substitution contexts. If after trimming, a MA had less than 10 columns, it was removed from the analysis. For the derivation of euarchonta- and glire-specific substitution rates, the MA data were bipartitioned accordingly and each part was pre-processed independently as above using the appropriate subset of trimming conditions.

**Evolutionary model estimation**

Strand-symmetric context-dependent substitution rates and branch lengths were estimated using Ambiore and PhyloFit [1,2]. The topology of the phylogenetic tree for euarchontoglires was taken from the 44-species UCSC conservation track of the human genome [3]. The initial branch lengths required for the subsequent application of Ambiore were determined with the Phylip DNAml module (<http://evolution.genetics.washington.edu/phylip.html>) [4], applied to a set of 10000 randomly chosen concatenated trimmed alignment blocks. The branch lengths of the resulting tree were scaled 200x and the initial model parameters were estimated from the concatenation of all trimmed alignment blocks, following the instructions in the Ambiore documentation.

Ambiore returns estimated mean substitution rates, branch lengths and 95% confidence intervals (1.96x standard deviation) for both, based on Bayesian Markov Chain Monte Carlo (MCMC) sampling. Whenever a cumulative result for a set of substitutions or branch lengths are reported here, it is the sum of the constituent mean values with the 95% confidence interval approximated by 1.96x the square root of the sum of the constituent variance values. PhyloFit does not estimate confidence intervals.

Ambiore and PhyloFit were applied to the same pre-processed MA data for each of the four sequence categories (P, M, L, HI). For each category, trimmed MAs (plus triple-columns of N at both ends) were concatenated into one large MA and used in the analysis. All available trimmed MAs for P and M were used, while for L and HI, 1000 each were randomly chosen. The MA data statistics are summarized in the following table.

| Category | #6kb regions | #Non-overlapping regions | #Untranscribed portions  = #MAs | #Trimmed MAs (euarchontoglires,  euarchonta only,  glires only) | #non-N columns in final MA  (euarchontoglires,  euarchonta only,  glires only) |
| --- | --- | --- | --- | --- | --- |
| P | 2 | 2 | 4 | 3, 3, 3 | 3773, 10994, 3720 |
| M | 13 | 13 | 82 | 57, 68, 57 | 26819, 46568, 26581 |
| L | 3602 | 3530 | 16019 | 11749, 14112, 11912 | 612226, 1039620, 602018 |
| HI | 11896 | 10872 | 57454 | 43606, 50362, 44211 | 575807, 962799, 609783 |

The number of Ambiore MCMC samples after the model parameter burn-in phase was set to 10000 for category P and 50000 for all other categories. Ambiore was run with the default root and rate category definitions and a single mutation type, except for the estimation of mutation type-specific branch lengths for which seven mutation types were defined, as in [1]. The run parameters for PhyloFit were the U3S substitution model (trinucleotide context-dependent symmetric non-reversible), non-overlapping tuples enabling use of the efficient expectation maximization algorithm, and medium convergence precision. The results obtained with PhyloFit and the U3S model were qualitatively and, with appropriate scaling, quantitatively similar to the Ambiore estimates, despite U3S not being equivalent to the Ambiore substitution model (U3S not only allows uvw->uv'w substitutions but also uvw->u'vw and uvw->uvw'). PhyloFit branch lengths were generally 3x the Ambiore branch lengths, while for uvw->uv'w substitutions modelled by both PhyloFit and Ambiore, the PhyloFit rates were a third of the Ambiore rates. Where Ambiore and PhyloFit estimates were directly and quantitatively compared, the PhyloFit values were scaled accordingly.

**Generation of MP, 0P and 00 embryos**

Wild-type and *Dnmt3L*^-/-^ female mice were induced to superovulation by i.p. injection of 5 IU PMSG followed 45-47h later by 5 IU hCG. Biparental embryos (MP) and maternal imprint-free (0P) embryos were isolated at the 1-cell stage after *in vivo* fertilization. Complete-imprint free (00) embryos were obtained by parthenogenetic activation of *Dnmt3L-/-* oocytes. Practically, metaphase II oocytes were released from the oviducts 13-16h following hCG injection and artificially activated in Ca^2+^-free KSOM medium containing 10mM SrCl2 (Sr^2+^) for 2h [5]. The second polar body extrusion was inhibited with 5ug/ml of cytochalasin B added during the strontium activation and during the next following 4h in KSOM^AA^ medium. Activated diploid oocytes were selected by the presence of a single polar body and two pronuclei. MP, 0P, and 00 zygotes were cultured in KSOM^AA^ medium in 5% CO_2_ at 37°C for 4 days, then transferred blindly to the uterine horns of Swiss Webster females on day 3 of pseudo-pregnancy. The day of transfer was recorded as 2.5dpc for the transplanted embryos. The number of transferred embryos was 90 for MP, 110 for 0P and 245 for 00. Implantation rates were respectively of 21, 34 and 43%. Twenty 0P and 00 embryos were compared at the phenotypic level.

**Gene expression analyses**

Total RNA was extracted using Trizol reagent (Invitrogen). Probes used for Northern blot hybridization were generated by PCR and are available upon request. cDNAs were synthesized using the Superscript II enzyme (Invitrogen) and used for real time expression quantification (7500 Real Time PCR system Applied Biosystems) using a SYBRGreen reaction mixture (SYBR GREEN PCR Master Mix, Applied Biosystems).

Amplifications were conducted using the following primers, whose products where all spanning introns: *Kcnq1ot1*, Kcnq1ot1-F 5’-AGG GCC AGG CCC ACT AGT-3’ and Kcnq1ot1-R 5’-ACC TGG ACA GCA GCC TGA GT-3’; *Cdkn1c*, Cdkn1c-F 5’- GAC GAT GGA AGA ACT CTG GG-3’ and Cdkn1c-R 5’-AGC GTA CTC CTT GCA CAT GG-3’; *Airn*, Airn-F 5’-GTG GAT TCA GGT TTC ATG-3’ and Airn-R 5’-GGC CCA GAT ATA GAA TGT-3’; *Igf2r*, Igf2R-F 5’-TAG TTG CAG CTC TTT GCA CG-3’ and Igf2R-R 5’-ACA GCT CAA ACC TGA AGC G-3’; *Gtl2*, Gtl2-F 5’-GGG CGC CCA CAG AAG AA-3’ and Gtl2-R 5’-GGT GTG AGC CGA TGA TGT CA-3’; *Dlk1*, Dlk1-F 5’-TTA CCG GGG TTC CTT AGA GC-3’ and Dlk1-R 5’-TGC ATT AAT AGG GAG GAA GGG-3’; *H19*, H19-F 5’-TTG CAC TAA GTC GAT TGC ACT-3’ and H19-R 5’-GGA ACT GCT TCC AGA CTA GGC-3’; *Igf2*, Igf2-F 5’-TTG TGC TGC ATC GCT GCT TAC-3’ and Igf2-R 5’-TAG ACA CGT CCC TCT CGG ACT T-3’; *beta-actin*, beta-actin-F 5’-AAG TGA CGT TGA CAT CCG-3’ and beta-actin-R 5’-GAT CCA CAT CTG CTG GAA GG-3’. Data were analyzed by normalizing the expression of imprinted genes to the level of expression of *beta-actin* for each sample and plotted by calibrating to the expression level in MP embryos.

### Methylation analyses

Bisulfite sequencing was performed on *in vitro* cultured 4.5dpc blastocysts and on 8.5dpc VYS according to Bourc’his *et al*., 2001 [6]. Primers for the analysis of the *H19* ICR (known as H19 DMD), the *Kcnq1ot1* ICR (KvDMR), the *Peg3* and *Snrpn* ICR were published [7,8,9]. Probes for the detection of LINE-1 (Type A) 5’-UTR and IAP LTR methylation by DNA blot hybridization were previously described [6].

**Determination of probe sets for the gene ontology analysis of developmental pathways affected by maternal and paternal methylation imprints**

For the determination of GO terms that were significantly affected by the absence of maternal imprints, each of the 19, 767 microarray probe sets that passed the above initial set of filters was assigned a score. If p(0P versus MP) ≤ 0.003 AND p(00 vs MP) ≤ 0.003 OR p(0P vs MP) ≥ 0.997 AND p(0P vs MP) ≥ 0.997, AND p(00 vs 0P) > 0.003 AND p(00 vs 0P) < 0.997 then score = max(GC-RMA(0P vs MP), GC-RMA(0P vs MP)), otherwise score = 0. In this model, p(A versus B) refers to the GCOS/MAS5-computed change p-value for the respective probe set when comparing arrays A and B. GC-RMA(A vs B) is the ratio, on a log_2_ scale, between the GC-RMA-computed [10] absolute expression levels that were measured by the probe set in samples A and B. The conditions on the change p-values ensure that the score is greater than zero only if the probe set detected significant differential expression in both 0P vs MP and 00 vs MP, but not in 00 vs 0P. This corresponds to the expected behavior of a gene that is regulated by maternal germline methylation.

When considering the impact of a lack of paternal imprints, the score definition analogously reflects the expected behavior: If p(00 vs MP) ≤ 0.003 AND p(00 vs 0P) ≤ 0.003 OR p(00 vs MP) ≥ 0.997 AND p(00 vs 0P) ≥ 0.997, AND p(0P vs MP) > 0.003 AND p(0P vs MP) < 0.997 then score = max(GC-RMA(00 vs MP), GC-RMA(00 vs 0P)), otherwise score = 0.

**References**

1. Hwang DG, Green P (2004) Bayesian Markov chain Monte Carlo sequence analysis reveals varying neutral substitution patterns in mammalian evolution. Proc Natl Acad Sci U S A 101: 13994-14001.

2. Siepel A, Haussler D (2004) Combining phylogenetic and hidden Markov models in biosequence analysis. J Comput Biol 11: 413-428.

3. Murphy WJ, Eizirik E, O'Brien SJ, Madsen O, Scally M, et al. (2001) Resolution of the early placental mammal radiation using Bayesian phylogenetics. Science 294: 2348-2351.

4. Felsenstein J (1989) PHYLIP- Phylogeny Inference Package (Version 3.2). Cladistics 5: 164-166.

5. O'Neill GT, Rolfe LR, Kaufman MH (1991) Developmental potential and chromosome constitution of strontium-induced mouse parthenogenones. Mol Reprod Dev 30: 214-219.

6. Bourc'his D, Xu GL, Lin CS, Bollman B, Bestor TH (2001) Dnmt3L and the establishment of maternal genomic imprints. Science 294: 2536-2539.

7. Tremblay KD, Duran KL, Bartolomei MS (1997) A 5' 2-kilobase-pair region of the imprinted mouse H19 gene exhibits exclusive paternal methylation throughout development. Mol Cell Biol 17: 4322-4329.

8. Yatsuki H, Joh K, Higashimoto K, Soejima H, Arai Y, et al. (2002) Domain regulation of imprinting cluster in Kip2/Lit1 subdomain on mouse chromosome 7F4/F5: large-scale DNA methylation analysis reveals that DMR-Lit1 is a putative imprinting control region. Genome Res 12: 1860-1870.

9. Lucifero D, La Salle S, Bourc'his D, Martel J, Bestor TH, et al. (2007) Coordinate regulation of DNA methyltransferase expression during oogenesis. BMC Dev Biol 7: 36.

10. Wu Z, Irizarry RA (2005) Stochastic models inspired by hybridization theory for short oligonucleotide arrays. J Comput Biol 12: 882-893.
